# Maternal willingness to participate in research involving neuroimaging, biological sample collection, and data storage: Towards a Multicentre Neurodevelopmental Research in a low-resource setting

**DOI:** 10.1371/journal.pone.0344408

**Published:** 2026-09-24

**Authors:** Albert Dayor Piersson, Christiana Amartey, Sarah Teiko Quartei, Klenam Dzefi-Tettey, Promise Emmanuel Sefogah, Aquel Rene Lopez

**Affiliations:** 1 York St John University, School of Science, Technology & Health, Department of Diagnostic Radiography, Lord Mayor’s Walk, York, North Yorkshire, England; 2 Centre for Education, Population Health Research & Innovation, Accra, Greater Accra Region, Ghana; 3 Sonotech Medical and Diagnostic Centre, Adjacent NIB Bank, Tema Community, Accra, Ghana; 4 University of Health and Allied Sciences, School of Medicine, Ho, Volta Region, Ghana; 5 University of Ghana Medical School, Dept of Obstetrics and Gynaecology, College of Health Sciences, Accra, Greater Accra Region, Ghana; 6 Tetteh Quarshie Memorial Hospital, Akuapem-Mampong, Eastern Region, Ghana; Yale University, UNITED STATES OF AMERICA

## Abstract

**Background:**

Maternal participation in neurodevelopmental research involving neuroimaging and diverse biological samples is essential for understanding prenatal influences on early brain development, yet willingness in low-resource settings remains underexplored.

**Method:**

We surveyed 300 mothers using a structured questionnaire to assess willingness to undergo brain health testing (with a focus on electroencephalography [EEG] and brain magnetic resonance imaging [MRI]), provide biological samples (blood, stool, urine, breast milk, placenta, amniotic fluid, vaginal/nasal fluid, saliva, tears), and consent to 10-year storage of diagnostic images and samples for future research. Responses were analysed to examine associations between maternal sociodemographic factors and willingness to consent for each research component.

**Results:**

Ninety-two percent of participants expressed willingness for brain health testing, including ~82% and ~88% interest in EEG and MRI, respectively, even for untreatable conditions. Self-reported histories of foetal defects (5.3%) and birth defects (7.3%) were notably low. Biospecimen acceptance was highest (>95%) for routine samples (blood, stool, urine) but significantly low for sensitive specimens (breast milk, placenta, amniotic fluid: 51–55%) including (vaginal fluid, saliva, tears: 16–47%). Higher levels of maternal education consistently predicted consent across modalities, while being married increased willingness for stool, urine, placenta, amniotic fluid, MRI, and EEG. Low income reduced uptake for placenta, amniotic fluid, MRI, and EEG. Only 48% consented to 10-year storage of diagnostic images and samples for future research.

**Conclusion:**

This study demonstrates high maternal willingness for neurodevelopmental research involving brain health testing and routine biospecimens in a low-resource setting. The findings highlight the feasibility of such protocols in a low-resource setting while exposing persistent inequities that risk underrepresenting disadvantaged mothers in maternal-child brain research. Contextually tailored consent models and capacity-building initiatives will be essential to ensure equitable, sustainable engagement across diverse LMIC populations.

## 1. Introduction

During gestation, factors such as maternal nutrition, stress hormones, immune status, and exposure to environmental toxins can epigenetically and directly influence foetal neurogenesis, neuronal migration, synaptogenesis, and myelination [1–5]. Postnatally, maternal caregiving behaviours, breastfeeding, social support, and the provision of a stimulating environment continue to exert significant influence on synaptic plasticity of the child’s brain, the maturation of neural circuits [4] and long-term cognitive and behavioural outcomes. Understanding these prenatal and postnatal influences often requires advanced methodologies such as neuroimaging, cognitive testing, and testing of biological samples to elucidate how the biological, environmental, and behavioural influences shape the developing brain. Techniques such as magnetic resonance imaging (MRI) [6], electroencephalography (EEG) [7,8], and eye tracking [9] may offer unprecedented insight into the structural, functional, and molecular processes shaping the developing brain.

Maternal biological samples provide complementary windows on prenatal exposures, molecular pathways, and maternal–foetal interactions shaping neurodevelopment from gestation into early life. Maternal blood captures micronutrient and toxicant profiles, with elevated manganese reportedly associated with poorer early childhood outcomes and higher selenium linked to increased autism spectrum disorder (ASD) or attention-deficit/hyperactivity disorder risk [10,11]. The maternal gut microbiome, profiled via stool, appears to have a stronger influence on infant neurodevelopment than the infant’s own early microbiome, and maternal dysbiosis has been associated with increased risks of neurodevelopmental disorders [12,13]. Urinary biomarkers of environmental toxicants (e.g., bisphenols, metals) have been linked to deficits in gross motor and problem-solving skills [14,15].

Breast milk integrates maternal health, environmental exposures, and nutritional signalling, simultaneously supporting neurodevelopment and carrying potential neurotoxicants such as heavy metals [16,17]. Placental tissue, central to foeto-maternal nutrient and gas exchange, endocrine function, and immune modulation, offers critical insight into in utero programming of brain development [18,19]. Amniotic fluid, enriched in metabolites such as 3-hydroxybutyrate relative to maternal blood, provides a direct readout of foetal brain energy metabolism [20,21]. The vaginal microbiome constitutes another potential causal pathway, with early evidence that vaginal microbiota transfer may safely modulate neurodevelopment [22,23].

Additional non-invasive matrices extend this neurodevelopmental “biospecimen toolkit.” The nose-brain interface contributes to immune regulation and cerebrospinal fluid clearance, and is being targeted for intranasal neuropeptide delivery, including arginine vasopressin, in ASD trials [24,25]. Saliva, which broadly mirrors plasma composition, is emerging as a feasible biomarker source for ASD screening and etiological studies, facilitating recruitment of neurotypical controls [26,27]. Maternal tear fluid, rich in proteins, lipids, metabolites, nucleic acids, and electrolytes, reflects systemic and ocular inflammation, and infant tears have been shown to activate cortical regions, including the lateral occipital and pre/postcentral gyri on functional MRI [28–31].Prior work has mainly examined parental attitudes toward single procedures such as imaging or isolated biospecimen collection [32–36]. However, few studies have systematically explored factors shaping maternal consent for comprehensive, longitudinal brain health research beginning *in utero*, which requires integrated neuroimaging and biological sampling to elucidate how maternal factors influence offspring brain development. Here, we focused on sociodemographic characteristics because willingness to participate in research may be shaped by differences in socioeconomic circumstances, education, social support, health literacy, and previous exposure to healthcare and research. Understanding these differences is particularly important in maternal and child research in low-resource settings, where practical constraints, perceptions of research, and unequal access to health information and services may influence who is able and willing to participate. Identifying sociodemographic patterns in willingness can therefore help researchers design recruitment, consent, and participation strategies that are inclusive, culturally sensitive, and ethically robust strategies for sustained engagement from pregnancy through early childhood and minimise the risk of systematically excluding particular groups from maternal and child research.

## 2. Methods

### 2.1. Study design

This study employed a cross-sectional design to assess maternal interest in and willingness to consent to participate in research that involves neuroimaging and the provision of biological samples for testing, geared towards multicentre neurodevelopmental research, with a focus on identifying the demographic and socioeconomic factors that influence consent. The start date of the study was 15/10/2023 and end date was 25/02/2024.

### 2.2. Participants, recruitment, and sampling

We targeted 385 pregnant women and mothers of infants/young children using convenience sampling from antenatal clinics and postpartum units at a public tertiary facility in Ghana. Eligible participants were mothers aged ≥18 years, fluent in English or a local language with validated translations, capable of providing informed consent, and (for prenatal recruitment) carrying singleton pregnancies without known major foetal anomalies. We excluded those with severe cognitive impairment precluding consent or a history of substance abuse that could compromise data reliability.

The target sample size of 385 was determined using Cochran’s formula for survey research:


$$\begin{array}{c} \left[{\text{n}}_{\text{0}}\;=\;({\text{Z}}^{\text{2}}\;\times \text{ p }\times \;(\text{1}-\text{p}))\;/\;{\text{e}}^{\text{2}};\text{ Z}=\text{1}.\text{96},\text{ p}=\text{0}.\text{5},\text{ e}=\text{0}.\text{05}\right] \end{array}$$

We received responses from 300 women.

### 2.3. Data collection

Data was collected using a structured pretested questionnaire which included sociodemographics (age, relationship status, educational attainment, monthly income [GH₵], *health insurance subscription*), and history of foetal or birth defect (Yes/No).

Maternal willingness to participate in research was assessed on the questionnaire by presenting hypothetical scenarios where participants were to indicate their willingness. We adopted questions 1 and 2 from a previous study [37]:

1. “Would you take a test even if it provides information about a disease that cannot be prevented or treated?”
2. “Imagine a simple brain health test to learn about risk of developing a brain disease. Would you wish to take such a test?”. We complemented this question with the options of two neuroimaging tests – Brain MRI and EEG and the following biological samples – blood, urine, breastmilk, nasal fluid, amniotic fluid, tears, saliva, vaginal fluid, stool, and placenta. Respondents were provided the options to select Yes, No, or Not sure.
3. We developed a question to assess willingness to allow the storage of your images/biological samples to be stored for at least 10 years for future research. Respondents were provided the options to select Yes, No, or Not sure.

#### 2.3.1. Questionnaire pretesting.

Before the main study, the questionnaire was pretested among a small group of pregnant women who met the study eligibility criteria but were not included in the final sample. The pretesting assessed the clarity, comprehensibility, relevance, and acceptability of the questions, as well as the time required for completion and the feasibility of the response options. Participants were encouraged to identify ambiguous, repetitive, or difficult-to-understand items, and their feedback was used to refine the wording, sequence, and formatting of the questionnaire. The revised instrument was subsequently reviewed by members of the research team with expertise in maternal health, neuroimaging, and research methodology to establish face and content validity. The pretesting process also supported assessment of the questionnaire’s internal consistency for multi-item constructs, with items demonstrating poor consistency or ambiguity reviewed and revised before administration in the main study.

### 2.4. Data analysis

All statistical analyses were performed using Real statistics. Age was reduced to young adults (18–30 years), middle-aged adults (31–40 years), and older adults (≥ 41 years). Relationship status was reduced to two variables as either “married” or “single” (widowed, divorced and single). We reduced highest education level to either “higher education” (professional training, higher national diploma, university) or “lower education” (none, primary school, junior high school, senior high school, vocational training). Then we stratified the monthly income status as follows: “low Income” (GHc0, 0–999), “middle Income” (1,000–1,999 and 2,000–2,999), or “high Income” (3,000–3,999 and ≥4,000). Frequencies and percentages were used to describe categorical variables, while means and standard deviations (or medians and interquartile ranges) were used to describe continuous variables. Responses to the questions on willingness to participate in research were dichotomised. Chi-square tests or independent t-tests were performed to examine associations between maternal sociodemographic factors and to participate to consent for each research component. Multivariable Logistic Regression models were constructed for each willingness to participate in research outcome to identify independent predictors. Maternal sociodemographic and health factors were entered as independent variables. Odds Ratios (OR) and 95% Confidence Intervals (CI) were reported. Statistical significance was set at *p* < 0.05.

### 2.5. Ethical consideration

The study protocol was approved by the Institutional Review Board of the University of Cape Coast (UCCIRB/EXT/2022/30). All participants were provided with detailed information about the study objectives, procedures, potential risks, and benefits, and were given the opportunity to ask questions before participation. Informed written consent was obtained from all participants before enrolment, with consent documented through signed consent forms in accordance with the approved ethical protocol and the principles of the Declaration of Helsinki. To maintain confidentiality, data were anonymised and used exclusively for research purposes. Emphasis was placed on the voluntary nature of participation and the right to withdraw at any time without penalty.

## 3. Results

Table 1 shows the descriptive statistics of participants’ sociodemographic characteristics.

**Table 1:** Sociodemographic characteristics of participants.

Respondents	n	% of total
Age range (years) 18–30 31–40 ≥ 41	205 82 13	68.3 27.3 4.3
Education Higher education Lower education	82 218	27.3 72.7
Relationship Single Married	50 250	16.7 83.3
Monthly income Low income Middle income High income	103 166 31	34.3 55.3 10.3
Subscription to health insurance Public Private None	274 22 4	91.3 7.3 1.3
Categories of participants Lactating Pregnant	172 128	57.3 42.7

Some total did not equal 100% due to rounding.

**Fig 1:**
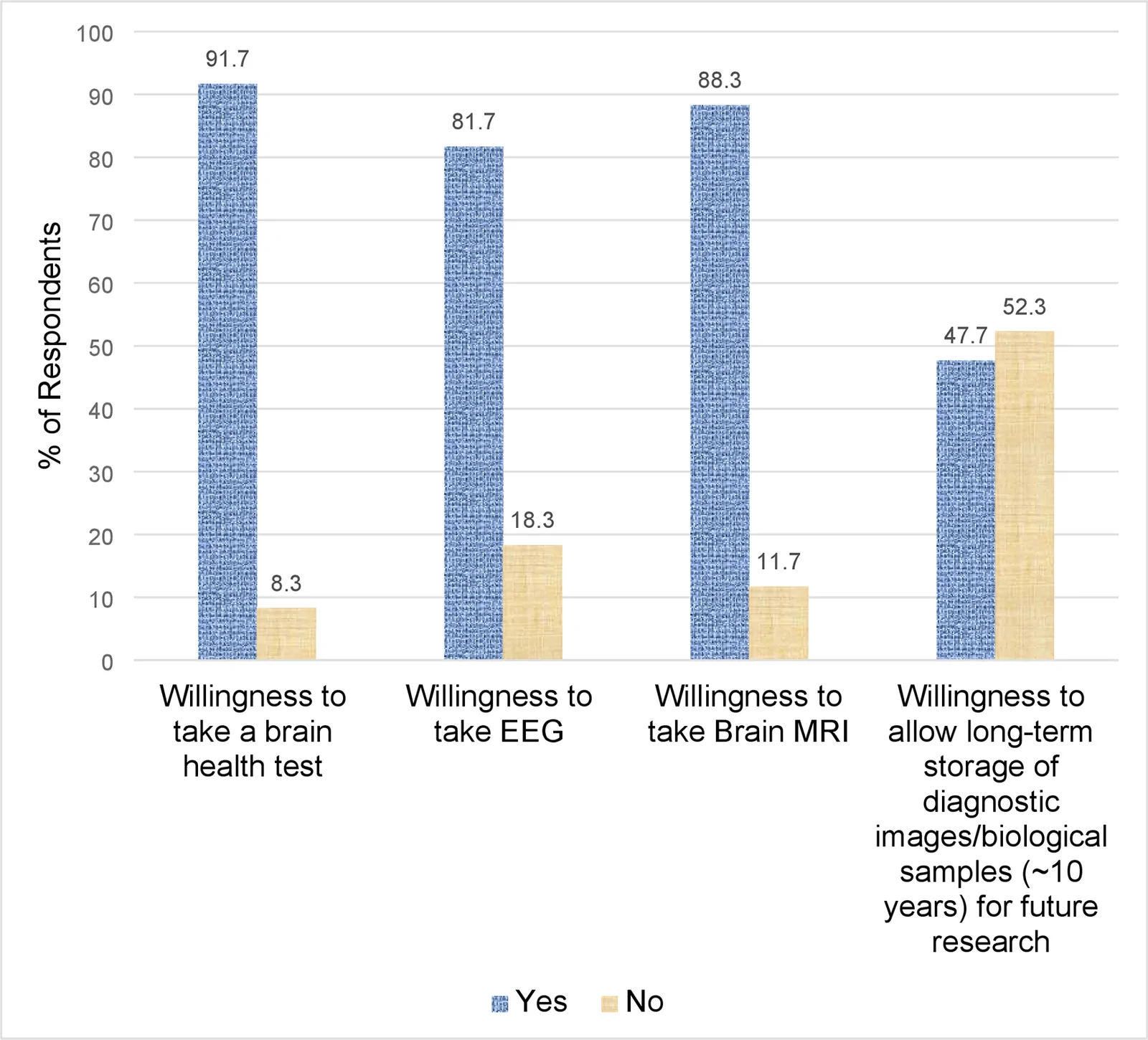
The graph plot shows responses on willingness to take a brain health test, brain imaging tests, and to allow long-term storage of diagnostic images/biological samples.

As shown in Fig 1, most participants (~92%) indicated their willingness to take a brain health test even if it provides information about a disease that cannot be prevented or treated. When asked if they would want to take brain imaging tests, **~**82% and **~**88% indicated they would take EEG and brain MRI, respectively.

### 3.1. Maternal willingness to take brain imaging and EEG tests

In response to whether participants would take a brain health test even if it provides information about a disease that cannot be prevented or treated, none of the sociodemographic factors predicted willingness to take these tests.

Being married was significantly associated with a higher likelihood of being willing to undergo brain MRI for the purposes of maternal and child neurodevelopmental research (β = 1.21, p = 0.006; OR = 3.35, 95% CI: 1.42–7.92). In contrast, being in a low-income bracket was significantly associated with a lower likelihood of willingness to undergo brain MRI for research purposes β = −1.22, *p* = 0.013; OR = 0.29, 95% CI: 0.11–0.77) (Table 2).

**Table 2:** Sociodemographic Predictors of Maternal Willingness to participate in research involving brain imaging and EEG tests.

Predictor Variable	β	SE	Wald	*p*-value	OR (95% CI)
Willingness to take Brain MRI					
Relationship status	1.21	0.44	7.61	.006	3.35 (1.42, 7.92)
Low income (vs Middle income)	−1.22	0.48	6.21	0.013	0.29 (0.11, 0.77)
Willingness to take EEG					
Age: ≥ 41 years	−1.50	0.70	4.57	0.033	0.22 (0.06, 0.88)
Relationship status	0.71	0.39	3.32	0.068	2.03 (0.95, 4.33)
Low income (vs Middle income)	−1.29	0.39	11.0	< 0.001	0.27 (0.13, 0.59)

In examining the willingness to take a brain health test (specifically EEG), women aged ≥41 years (β = −1.50, *p* = 0.033; OR = 0.22, 95% CI: 0.06–0.89) and those with low-income (β = −1.29, *p* < 0.001; OR = 0.27, 95% CI: 0.13–0.59) were less likely to take the test (Table 2). In contrast, those married (β = 0.71, *p* = 0.068; OR = 2.03, 95% CI: 0.95–4.33) were more likely to want to take the test, albeit marginally.

### 3.2. Association between having had previous foetal/birth defect history and willingness to take brain test

**Fig 2:**
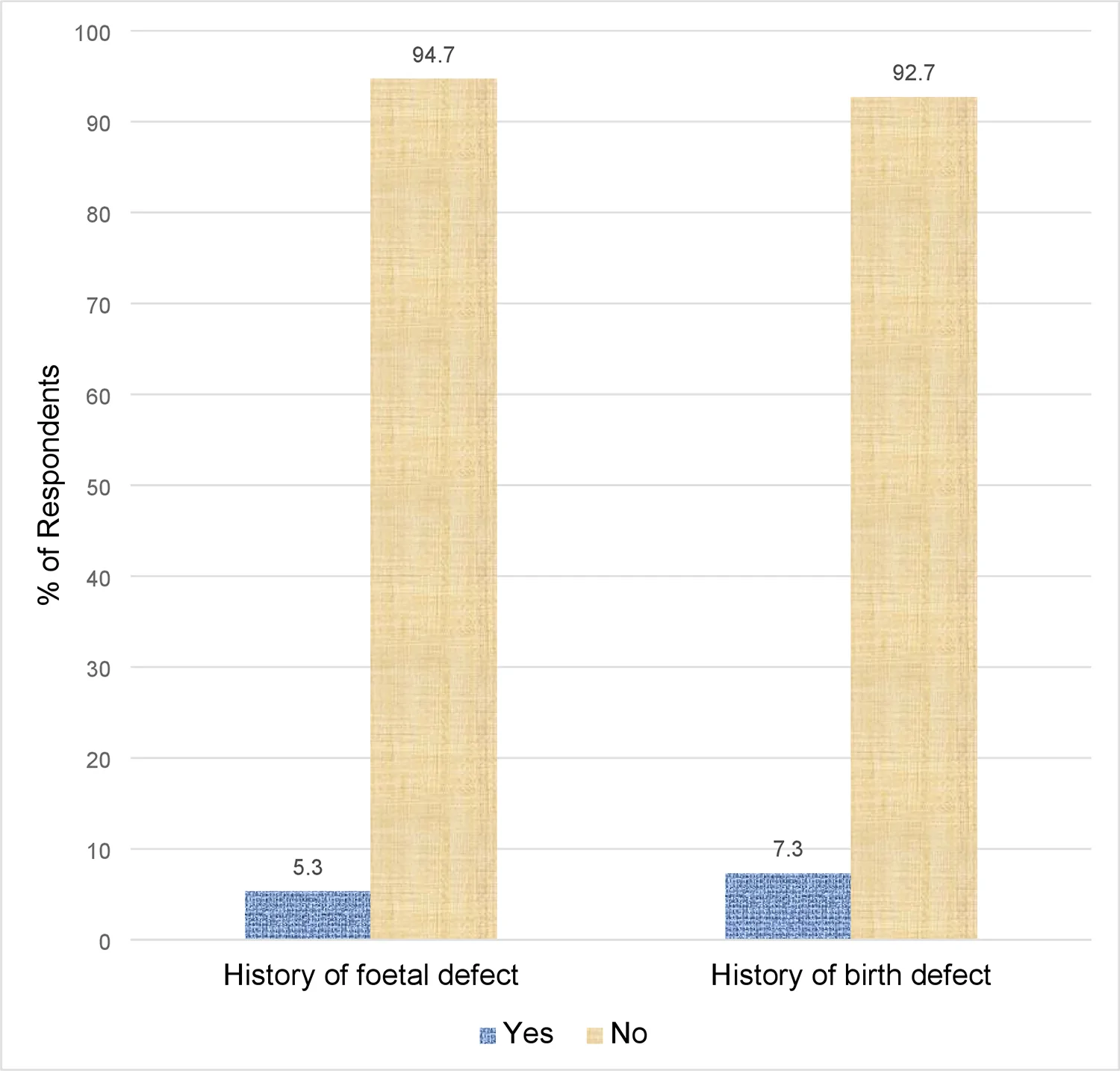
Plot shows maternal history of foetal/birth defect.

Fig 2 shows that only 5.3% indicated they had a history of foetal defect while 7.3% of the participants indicated they had history of birth defect.

We found no statistically significant association between having had a history of foetal defects and willingness to undergo brain health testing even if it provides information about a disease that cannot be prevented or treated (ꭓ^2^ = 0.10, *p* = 0.757), brain MRI (ꭓ^2^ = 0.48, *p* = 0.488) and EEG (ꭓ^2^ = 1.65, *p* = 0.200). In addition, we found no statistically significant association between having had a history of birth defects and willingness to undergo brain health testing even if it provides information about a disease that cannot be prevented or treated (ꭓ^2^ = 0.45, *p* = 0.504) including brain MRI (ꭓ^2^ = 1.17, *p* = 0.280) and EEG (ꭓ^2^ = 1.35, *p* = 0.244).

### 3.3. Willingness to provide biological samples

**Fig 3:**
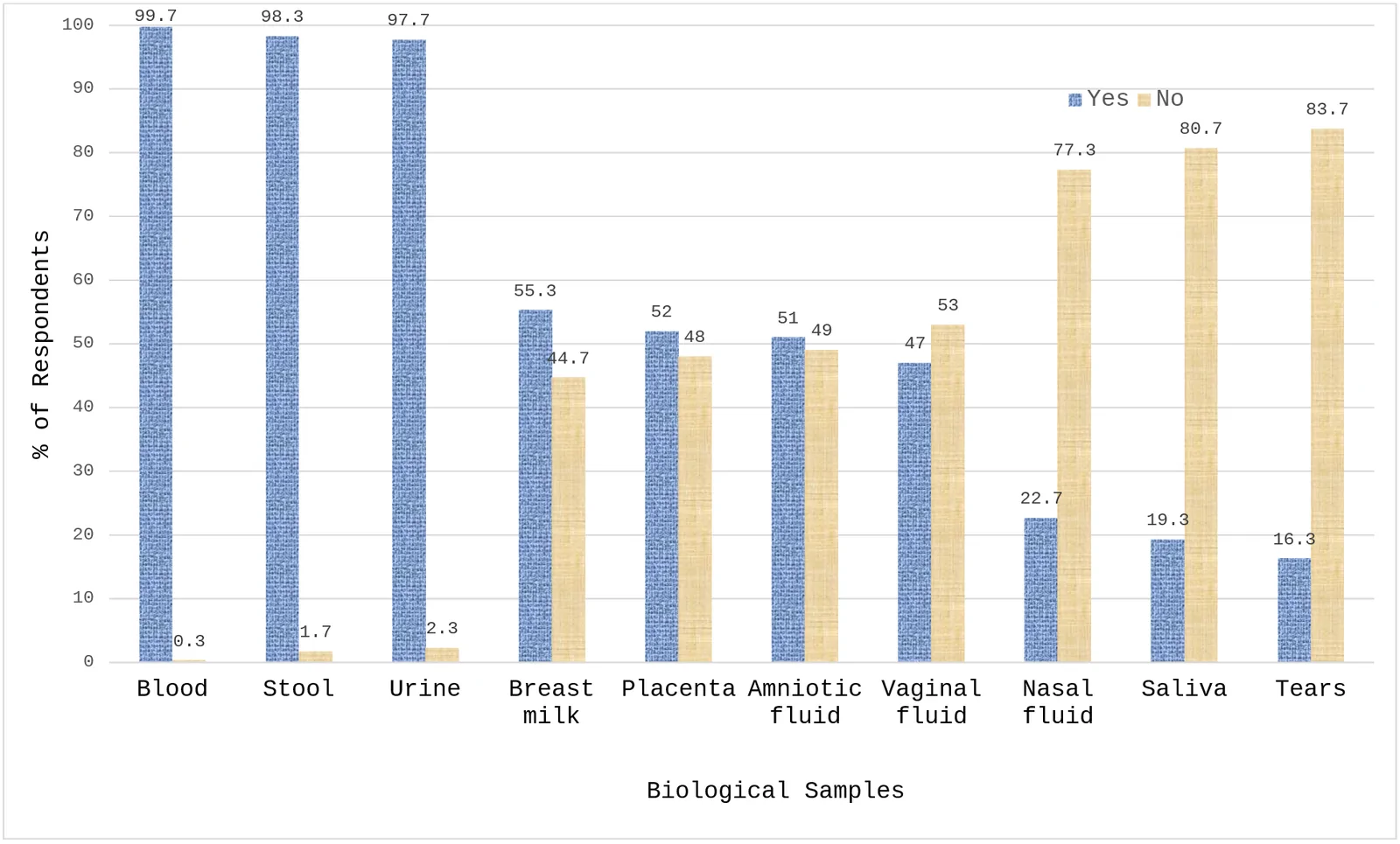
Plot shows willingness to provide biological samples.

Participants demonstrated high willingness to provide commonly collected biological samples, with 99.7% consenting to blood, 98.3% to stool, and 97.7% to urine Fig 3. Moderate willingness was observed for breast milk (55.3%), placenta (52%), and amniotic fluid (51%) while lower willingness was reported for less commonly collected samples, such as vaginal fluid (47%), nasal fluid (22.7%), saliva (19.3%), and tears (16.3%).

Maternal willingness to provide biospecimens varied substantially across specimen types (Table 3). No demographic or socioeconomic predictors were associated with willingness to provide blood. While relationship status and low-income showed marginal positive associations for stool, both were significant predictors of increased willingness to provide urine.

**Table 3:** Sociodemographic Predictors of Maternal Willingness to provide biological samples.

Predictor Variable	β	SE	Wald	*p*-value	OR (95% CI)
Stool					
Relationship status	1.88	1.08	3.03	0.082	6.56 (0.79, 54.53)
Low income (vs Middle income)	2.42	1.32	3.37	0.067	11.19 (0.85, 147.74)
Urine					
Relationship status	2.18	0.91	5.81	0.016	8.88 (1.50, 52.42)
Low income (vs Middle income)	2.71	1.25	4.71	0.030	15.10 (1.30, 175.21)
Breast Milk					
Higher education	1.78	0.44	16.26	< 0.0001	5.94 (2.50, 14.11)
Low income (vs Middle income)	−0.80	0.31	6.45	0.011	0.45 (0.24, 0.83)
Placenta					
Relationship status	0.91	0.46	3.97	0.046	2.48 (1.02, 6.07)
Higher education	2.0	0.47	18.06	< 0.0001	7.35 (2.93, 18.45)
Low income (vs Middle income)	−1.27	0.33	14.53	< 0.001	0.28 (0.15, 0.54)
Amniotic fluid					
Relationship status	1.06	0.48	4.93	0.026	2.89 (1.13, 7.39)
Higher education	1.95	0.46	18.27	< 0.0001	7.05 (2.88, 17.28)
Low income (vs Middle income)	−1.23	0.34	13.38	< 0.001	0.29 (0.15, 0.57)
Vaginal fluid					
Higher education	1.67	0.41	16.99	<0.0001	5.32 (2.40, 11.79)
Low income (vs Middle income)	−1.52	0.36	17.86	< 0.0001	0.22 (0.11, 0.44)
Nasal fluid					
Higher education	2.15	0.40	29.02	< 0.0001	8.62 (3.94, 18.87)
Lack of health insurance	2.28	1.27	3.22	0.073	9.82 (0.81, 110.27)
Saliva					
Higher education	2.13	0.42	25.73	< 0.0001	8.41 (3.69, 19.16)
Lack of health insurance	2.69	1.29	4.39	0.036	14.77 (1.19, 183.52)
Tears					
Higher education	2.67	0.50	29.0	< 0.0001	14.42 (5.46, 38.10)
Lack of health insurance	3.14	1.33	5.58	0.018	23.06 (1.71, 311.38)

Across breastmilk, placenta, amniotic fluid, vaginal fluid, nasal fluid, saliva, and tears, higher maternal education consistently emerged as the strongest positive predictor, but being married specifically predicted willingness to provide only placenta and amniotic fluid. However, low-income status was consistently associated with reduced willingness to provide these biological samples. Notably, lack of health insurance showed sporadic positive associations for willingness to provide nasal fluid, saliva, and tears.

### 3.4. Willingness to allow long-term storage of diagnostic images and biological samples

Slightly less than half of the participants (~48%) would allow long-term storage of their diagnostic images/biological samples for future research (see Fig 1).

In assessing response to the long-term storage of images or biological samples for future research (~10 years), higher educational attainment was a significant positive predictor (β = 1.47, *p* < 0.001, OR = 4.35, 95% CI: 2.04–9.24), with mothers possessing higher education levels being over four times more likely to consent (Table 4). Conversely, low-income status was a significant negative predictor (β = −1.19, *p* < 0.001, OR = 0.30, 95% CI: 0.16–0.59), with mothers in the low-income group being substantially less likely to consent compared to those with middle income.

**Table 4:** Sociodemographic predictors of maternal willingness to allow long-term storage of diagnostic images and biological samples.

Predictor Variable	β	SE	Wald	*p*-value	OR (95% CI)
Willingness to allow long-term storage of diagnostic images and biological samples					
Higher education	1.47	0.38	14.58	< 0.001	4.35 (2.04, 9.24)
Low income (vs Middle income)	−1.19	0.34	12.51	< 0.001	0.30 (0.16, 0.59)

## 4. Discussion

This cross-sectional study explored maternal interest and consent preferences regarding participation in neurodevelopmental research involving neuroimaging and biological sample collection, critical components for advancing multicentre investigations into *in utero* to early brain development. Our key finding revealed a strong public interest in brain health testing, even when the conditions being assessed are untreatable or cannot be prevented. These findings are discussed below.

### 4.1. Maternal Interest and sociodemographic predictors of willingness to take brain health test and participate in brain imaging and EEG tests

About 92% of participants were willing to participate in research involving brain health testing, even for untreatable conditions, while more than 80% were willing to participate in research involving MRI or EEG. Such openness suggests growing engagement with proactive brain health monitoring, consistent with earlier findings of strong public interest in neurodiagnostic testing [38–43]. Unlike studies linking willingness to demographic or socioeconomic factors [44], our data showed no such associations, pointing to broad, cross-group receptivity. Yet this apparent consensus may conceal anxiety about learning untreatable results, underscoring the need for clearer communication on the purpose and implications of neurodiagnostic testing [42,43].

When we focused on two specific neuroimaging tests (i.e., brain MRI and EEG), participants who were married were more willing to undergo brain MRI. This association may reflect the role of interpersonal and household support in facilitating decisions about research participation, particularly when participation involves unfamiliar procedures such as neuroimaging. Social support may therefore be particularly relevant for maternal neurodevelopmental research, where participation can involve additional considerations related to pregnancy, time, travel, and concerns about maternal and foetal safety. Conversely, participants from lower-income groups were less likely to be willing to participate in research involving brain MRI. This association may reflect structural barriers that extend beyond the direct cost of research participation. Lower-income women may face greater constraints related to awareness, transportation, time away from work or household responsibilities, childcare, and other indirect costs associated with attending research appointments. Several of these factors have been highlighted in previous studies [45–48]. Interestingly, older age (≥41 years) and low income were associated with reduced willingness to participate in EEG research, potentially reflecting differences in prior exposure to neurodiagnostic procedures, concerns about unfamiliar technology, and competing practical or financial demands that may make research participation less feasible. In settings where EEG and other neurodiagnostic services remain relatively limited, unfamiliarity may further contribute to uncertainty about what the procedure entails and its potential value, particularly among women with fewer opportunities to encounter such technologies [47, 48]. In contrast, women who were married showed slightly greater willingness, suggesting that supportive relationships may provide opportunities to discuss research, clarify concerns, and strengthen confidence in decisions about participation.

### 4.2. Association between maternal foetal/birth defect history and willingness to take brain test

We found that only 5.3% and 7.3% of participants reported histories of foetal and birth defects, respectively, broadly consistent with previous estimates from Ghana and similar settings [38–41,49]. The relatively low self-reported prevalence may reflect not only underdiagnosis and incomplete documentation, but also limited recognition of congenital and neurodevelopmental conditions and the stigma that can discourage families from disclosing such experiences. This context reinforces the importance of improving public awareness and health literacy to support earlier recognition and intervention [50]. Notably, a history of foetal or birth defects was not associated with willingness to participate in research involving brain health testing, including testing for untreatable conditions. This may suggest that personal experience with an adverse outcome may not necessarily translate into greater openness to research participation [44]. For some mothers, previous adverse experiences may be accompanied by emotional distress, concerns about receiving further unfavourable information, or uncertainty about the practical value of detecting conditions for which no treatment exists [50]. Conversely, limited understanding of the potential contribution of research to future maternal and child health may reduce willingness to participate. These findings suggest that recruitment should not assume that personal experience alone creates willingness, but should provide sensitive, clear explanations of the purpose of research, including how findings may contribute to knowledge and benefit future families even when immediate treatment is unavailable.

### 4.3. Maternal interest and sociodemographic predictors of willingness to provide biological samples

Over 95% of participants were willing to provide common biological samples (blood, stool, urine), reflecting their routine clinical use, familiarity, and perceived low risk. This corroborates our earlier findings [38–41] and that of others [51,52]. Importantly, maternal willingness to provide blood showed no demographic or socioeconomic associations. This is consistent with its status as a widely accepted, low-risk procedure, with needle phobia and anxiety likely exerting limited influence in this context [35,53,54]. Stool and urine provision were also higher than in many previous studies [55,56]. Though willingness for stool provision showed modest associations with being married and lower income, urine provision willingness was higher among partnered and lower-income mothers.

By contrast, willingness to provide breast milk, placenta, and amniotic fluid was moderate (about 51–55%), in line with our earlier work [38–41] but lower than some African studies on breast milk donation [57]. Despite the recognised neurodevelopmental value of breast milk [58,59], reported barriers, such as time constraints, dislike, limited knowledge or supply, infection fears, and cultural or religious beliefs together with concerns about commercialisation and exploitation of low-income women may have contributed to this lower uptake [60,61].

Higher maternal education and being married consistently predicted willingness to provide placenta and amniotic fluid; higher maternal education was also associated with willingness to provide breast milk. These associations may reflect differences in health and research literacy, confidence in understanding the purpose and potential benefits of biological sample donation, and the availability of social support when making decisions about participation. For breast milk and placental tissue, which are closely connected to pregnancy and motherhood, women with greater educational exposure may be better positioned to understand how these biological materials can contribute to knowledge about maternal and child health, while support from a spouse may provide reassurance when considering the collection and long-term use of biological samples. In contrast, low-income status reduced willingness to provide placenta for research. This raises concerns that specific biospecimen research in LMICs may systematically underrepresent socioeconomically disadvantaged mothers and perpetuate inequities in maternal–child health evidence. Although prior studies have demonstrated the feasibility of obtaining amniotic fluid for prenatal screening of adverse perinatal outcomes [62,63], our findings indicate moderate willingness to provide this sample, likely reflecting apprehension about its invasive nature. Socioeconomic disparities again influenced participation, with lower-income status associated with reduced willingness.

Willingness to provide less commonly clinically collected samples (i.e., vaginal and nasal fluid, saliva, and tears) was lower (approximately 16–47%), echoing our previous findings [38–41]. However, it contrasts with reports of high consent rates for vaginal samples in other African cohorts [36]. Reluctance toward vaginal fluid sampling has been linked to embarrassment, discomfort, and religious or cultural norms [36,64]. In our study, higher education mitigated this reluctance, whereas financial disadvantage exacerbated it. Further, we found that higher education and lack of health insurance (the later albeit marginally) were associated with increased willingness to provide nasal fluid. This highlights complex motivations in the context of emerging nose-to-brain therapeutics. Although both saliva and tear fluid are promising non-invasive biospecimens with growing roles in neurodevelopmental and proteomic research [65,66], we found that willingness to provide either of them was driven by higher education and lack of health insurance.

### 4.4. Sociodemographic predictors of maternal willingness to allow long-term storage of diagnostic images and biological samples

We proposed a 10-year retention period for diagnostic images and biological samples, consistent with reported Picture Archiving and Communication System retention practices ranging from 6 to 240 months [67,68]. Only 48% of participants consented to long-term storage of diagnostic images and samples for future research. This is consistent with prior work [38–41], highlighting the tension between the potential scientific value of retaining research materials and participants’ concerns about privacy, future use, and control over their information and samples. These concerns may be particularly salient in resource-limited settings, where uncertainties about the security of stored data and biological materials may coexist with infrastructural limitations that could affect long-term preservation and sample integrity [9,69]. We found that higher maternal education was associated with greater willingness to consent to long-term storage of both diagnostic images and biological samples. This is consistent with previous studies [70,71], potentially because greater health and research literacy may help participants understand why long-term retention can support future research and generate knowledge beyond the original study. This finding is also consistent with evidence that individuals may be willing to support data sharing despite recognising that secondary use can reduce their control over personal information [72,73]. Conversely, lower-income status was associated with reduced willingness to consent to long-term storage, which may reflect broader concerns about trust, perceived vulnerability, future misuse of information, and limited confidence in the institutions responsible for safeguarding stored materials [74]. Such differences are important because socioeconomic inequalities in willingness to permit long-term storage could ultimately shape whose data and biological materials are available for future research, potentially reinforcing existing inequities in evidence generation. It is important to highlight that our study specifically examined long-term storage consent, which differs conceptually from data-sharing preferences and warrants separate exploration, particularly in light of emerging artificial intelligence (AI)-driven research requiring secondary data reuse.

### 4.5. Strengths and limitations

To the best of our knowledge, this study is among the first to assess maternal willingness for neurodevelopmental research involving diverse biological samples, imaging modalities, and long-term storage of diagnostic images/biological samples in a low-resource setting, offering insights for ethical recruitment strategies in our setting.

Our study is also not without some limitations. This is a cross-sectional study which focused on a single region and involved only maternal (not paternal) samples, with a modest sample size limiting generalisability. Potential social desirability bias cannot be completely excluded. Finally, participant responses were based on hypothetical willingness, which may differ from decisions made when faced with actual research procedures, especially for more invasive sampling. Therefore, stated willingness may overestimate actual participation when faced with the practical, emotional, financial, or logistical demands of research participation. Nevertheless, we sought to maximise the ecological validity of participants’ responses by providing clear and study-specific information about the proposed procedures, including neuroimaging, biological sample collection, data storage, and potential risks and benefits, and by assessing willingness across these individual research components rather than using a single global question. The questionnaire was also pretested to ensure that the scenarios and response options were understandable and appropriately reflected the proposed research context. Thus, the findings should be interpreted as measures of stated willingness and acceptability, rather than direct evidence of actual research enrolment.

## 5. Conclusion and future directions

This study reveals high maternal willingness for routine biospecimens (blood, urine, stool) and brain health testing (MRI, EEG), but lower acceptance for sensitive samples (breast milk, placenta, vaginal fluid, tears) and to permit long-term storage, with educational level consistently predicting participation and socioeconomic/relational factors shaping uptake. Understanding these sociodemographic differences is important for maternal research because women who face educational or social barriers may be less likely to contribute valuable biological specimens, potentially creating selection bias and limiting the representativeness of research findings. Furthermore, the relatively low willingness to permit long-term storage of images and biological samples, suggests that the scientific value of longitudinal and secondary-use research must be balanced against participants’ concerns regarding privacy, future use, ownership, and loss of control. These patterns also raise an important representation concern: if women with lower educational attainment, lower income, or less social support are disproportionately less willing to provide sensitive specimens or permit long-term storage, future biobanks and neurodevelopmental datasets may systematically underrepresent socially and economically disadvantaged mothers. This may limit the generalisability and equity of research findings, highlighting the need for ethically sensitive, culturally appropriate protocols in low-resource neurodevelopmental research setting.

We also highlight that our quantitative design cannot establish the underlying reasons for the differences elicited in our study. In particular, we did not collect qualitative data or explicitly assess experiences of discrimination, structural barriers, or marginalised identities. In this regard, we cannot determine the extent to which these factors contributed to observed willingness rates. This is an important direction for future research, particularly in LMIC settings where social, economic, cultural, institutional, and historical factors may interact to shape trust and research participation. Mixed-methods and community-engaged approaches could provide greater insight into why women accept or decline specific forms of biological sampling and long-term data retention. Such approaches could also support the development of culturally appropriate consent processes, address practical and informational barriers, and help ensure that advances in maternal and child neurodevelopmental research do not inadvertently reproduce existing inequities. Importantly, recruitment and consent strategies may consider the use of accessible explanations of the purpose, handling, and potential future value of biological samples and provide opportunities for participants to discuss concerns and make informed decisions independently. Future multicentre longitudinal studies may consider the integration of parental sociodemographic, clinical, biological, environmental, and neuroimaging data with repeated assessments of maternal and child outcomes to elucidate mechanisms linking maternal health, prenatal exposures, and neurodevelopmental. Where feasible, integration of biological specimens, genetic or molecular biomarkers, and detailed measures of social and environmental determinants could further clarify underlying mechanisms and identify modifiable risks and protective factors. Such multimodal longitudinal datasets could also support the development and validation of predictive models for early identification of children at increased risk of adverse neurodevelopmental outcomes, while enabling assessment of how these pathways vary across socioeconomic and cultural contexts. Ultimately, establishing contextually grounded ethical frameworks and community engagement strategies will be pivotal in supporting large-scale, sustainable maternal-child brain health research across diverse populations in LMICs.

### Open access statement

For the purpose of open access, the author(s) has applied a Creative Commons Attribution (CC BY) licence to any Author Accepted Manuscript version arising from this submission.

### Declaration

Parts of the findings reported in this manuscript were previously presented at the following scientific meetings: the *2024 European Society of Radiology (ESR) Congress*, Austria; the *2024 York St. John University Conference—“The Only Way Is Ethics! Reflecting on and Learning from Research After Ethical Approval”* (published in the conference proceedings); the *2024 European Society for Magnetic Resonance in Medicine and Biology (ESMRMB) Congress*, Spain; and the *2024 Neonatal Society Autumn Meeting*, United Kingdom.
